# Comparative Effects of Er-Xian Decoction, *Epimedium* Herbs, and Icariin with Estrogen on Bone and Reproductive Tissue in Ovariectomized Rats

**DOI:** 10.1155/2012/241416

**Published:** 2012-11-07

**Authors:** Liming Xue, Yin Wang, Yiping Jiang, Ting Han, Yan Nie, Lu Zhao, Qiaoyan Zhang, Luping Qin

**Affiliations:** ^1^School of Pharmacy, Second Military Medical University, No. 325 Guohe Road, Shanghai 200433, China; ^2^Department of Pharmaceutics, No. 455 Hospital of CPLA, Shanghai 200052, China; ^3^Department of Pharmacy, Fujian University of Traditional Chinese Medicine, Fuzhou 350108, China

## Abstract

Er-Xian Decoction (EXD), *Epimedium* herbs (herbs of *Epimedium brevicornum* Maxim, EBH), and icariin (ICA) have been proven to have estrogen-like and antiosteoporotic activity and are used for the treatment of osteoporosis, menopausal syndrome, and age-associated diseases. The present study found that EXD, EBH, and ICA treatments, emulating estrogen, significantly contributed to bone density and architecture in OVX rats and that EXD is similar to estrogen and exerts a concomitant effect on bone formation and bone resorption at the tissue level, while EBH and ICA produced bone-protective effects mainly by inhibiting bone resorption. Nevertheless, EXD, EBH, and ICA treatments manifested a fewer adverse effects on the uterus, mammary gland, and vagina compared to estrogen administrations. Among the EXD, EBH, and ICA, EXD was found to have superior efficacy and safety profile.

## 1. Introduction

Postmenopausal osteoporosis is a common disease characterized by a systemic impairment of bone mass and microarchitecture that result in fragility fractures [1]. As gonadal hormone deficiency plays a key role in the pathogenesis of postmenopausal osteoporosis, estrogen replacement therapy was regarded in past decades as the most effective and the first choice for the treatment of this disease [2]. Estrogen regulates bone remodeling via estrogen receptor alpha (ER*α*) and estrogen receptor beta (ER*β*). The uterus, vagina, and mammary gland express estrogen receptor and are targets for estrogen. Therefore estrogen may exert stimulatory effects on these estrogen-sensitive organs. Also, estrogen exposure enhances the expression of progesterone receptor (PR), through which progesterone antagonizes the stimulatory effects of estrogen on endometrial tissue [3]. The Women's Health Initiative enrolled more than 150,000 women for a comprehensive trial regarding the effect of combined estrogen and progestin. The risk-benefit index highlighted coronary heart disease, invasive breast cancer, pulmonary embolism, endometrial cancer, and colorectal cancer as side effects [4, 5]. Since the overall health risks exceeded benefits, the trial was stopped. Therefore, the interest to find effective and safe alternatives for the treatment of osteoporosis has grown in recent years [4].

Traditional Chinese medicines containing multi-interactive compounds, which have been used for centuries in China for treatment of bone disorders, have attracted the attention of researchers for their effects on the management of menopausal and related medical conditions [6]. Er-Xian Decoction (EXD), a popular Chinese medicinal formula comprised of *Epimedium brevicornum *Maxim (Berberidaceae, whole herb, EBH), *Curculigo orchioides *Gaertn. (Amaryllidaceae, rhizome, COR), *Anemarrhena asphodeloides *Bge. (Liliaceae, rhizome, AAR), *Phellodendron chinense *Schneid (Rutaceae, bark, PCB), *Morinda officinalis *How. (Rubiaceae, root, MOR), and *Angelica sinensis *(Oliv.) Diels (Umbelliferae, root, ASR) in a compositional ratio of 9 : 9 : 6 : 6 : 9 : 9, has been used for the treatment of osteoporosis, menopausal syndrome, and age-associated diseases over the past 60 years [7]. A systemic review and meta-analysis of 677 participants involved in 5 clinical investigations indicated that EXD was clinically effective in relieving menopausal syndrome via increasing circulatory estradiol levels [8, 9]. Both animal experiments and clinical practices proved that EXD had definite protective effects on bone loss induced by estrogen deficiency [7, 10, 11].

According to traditional Chinese medicine, *Epimedium brevicornum *and *Phellodendron chinense* are essential ingredients of EXD and appear to play important roles in pmeliorating signs and symptoms of the menopausal syndrome and osteoporosis. *Curculigo orchioides* and *Morinda officinalis* help strengthen the curative effect of the *Epimedium brevicornum*, and *Anemarrhena asphodeloides* helps *Phellodendron chinense* to play a fuller role. *Angelica sinensis *is another ingredient that cooperates with the above five medications to strengthen the therapeutic effects and treat the accompanying disease or syndromes. *Epimedium* herb, as an essential ingredient of EXD, has been reported to possess phytoestrogen-like flavonoids including icariin, incaritin, and baohuoside. These phytoestrogen flavonoids have been shown to exert protective effects on bone loss in OVX rats and postmenopausal women [12, 13]. However, it has been demonstrated that *Epimedium* flavonoids are weak estrogen agonists that act via the ER*α* and ER*β* [14]. Phytoestrogens have the ability to interfere with estrogen action either by interacting directly with the estrogen receptor (ER) or indirectly by modulating endogenous estrogen concentrations [15, 16]. Accumulating evidence from *in vitro* experiments, animal studies, and human clinical trials suggests that in addition to their beneficial effects, phytoestrogens may also increase the risk for several hormone-dependent diseases in humans [17]. Therefore, systemic treatment with EXD, *Epimedium* herbs, or icariin can be expected to exert weak estrogen agonistic effects on estrogen-receptor-positive tissues and may produce unwanted hormone-related side effects such as endometrial hyperplasia and endometrial carcinomas.

The aim of the present study was to compare with estrogen the bone protective effects of EXD, *Epimedium* herbs (EBH), and icariin (ICA) in ovariectomized rats. The safety of EXD, EBH, and ICA was also determined by observing the histologic structure changes and measuring the ER*α*, ER*β*, and PR expressions in reproductive tissues.

## 2. Materials and Methods

### 2.1. Materials

The assay kits for alkaline phosphatase (ALP), tartrate resistant acid phosphatase (TRAP), serum or urine calcium (Ca), inorganic phosphorus (P), and urine creatinine (Cr) were purchased from Nanjing Jiancheng Bioengineering Institute (Nanjing, China). Radioimmunoassay (RIA) kits for measurement of estradiol levels were purchased from China Institute of Atomic Energy (Beijing, China). Nylestriol was purchased from Hualian Pharmaceutical Company (Shanghai, China,). Icariin (ICA) and extracts of *Epimedium *herbs (EBH) were purchased from Huike Biopharmaceutical Company (Xi'an, China). The six plant materials in EXD were obtained from Hua Yu Pharmaceutical Company (Shanghai, China) and identified by Professor H.C. Zheng; the voucher specimen numbers for EBH (2010110601), COR (2010110602), AAR (2010110603), PCB (2010110604), MOR (2010110605), and ASR (20101106) are available at the herbarium of the Department of Pharmacognosy, School of Pharmacy of the Second Military Medical University (Shanghai, China). The six plant materials were mixed in a compositional ratio of 9: 9: 6: 6: 9: 9 according to the compatible theory in traditional Chinese medicine (EBH: 281.25 g, COR: 281.25 g, AAR: 187.5 g, PCB: 187.5 g, MOR: 281.25 g, and ASR: 281.25 g; dried materials) and extracted by decocting the mixed herbs with 10x (v/w) distilled water at 100°C for 2 hours. After filtration, the residue was boiled for an additional 1 hour. Filtrates were mixed together and lyophilized with a freeze drier (Labconco, FreeZone), and the resulting 150 g of dry powder were kept at 4°C.

### 2.2. Animals and Treatments

Forty-eight female Sprague-Dawley (SD) rats aged 12 weeks were purchased from Slacom Experimental Animal Company (Shanghai, China, SCXK: 20070003) and acclimated to conditions for 1 week before the experiment. The experimental animals were housed in an air-conditioned room with 12 h/12 h light-dark illumination cycles at constant temperature (25 ± 2°C) and humidity (50% ± 10%). Food and drinking water were supplied ad libitum. The rats were weighed weekly during the experimental period. The osteoporotic model was established 12 weeks after bilateral ovariectomy. Of the 48 female SD rats, 8 were sham operated and treated with deionized water as the control group (sham). The remaining rats were bilaterally ovariectomized and equally randomized into five groups. Rats were treated with deionized water (OVX model control), NYL (Nylestriol, 1 mg/kg, weekly), ICA (Icariin, 20 mg/kg, daily), EBH (extracts of *Epimedium *herbs, 100 mg/kg, daily), or EXD (Er-Xian Decoction; 600 mg/kg, daily) by intragastric administration for 12 weeks. This experiment was approved by the Bioethics Committee of the Second Military Medical University (Shanghai, China), and the procedures of the experiment strictly adhered to generally accepted international rules and regulations.

### 2.3. Sample Collection

At the end of treatment, the rats were housed individually in metabolic cages without food for 1 day. Urine samples were collected from 20:00 pm to 8: 00 am the next day. Blood samples were collected via the abdominal artery, stabilized with sodium heparin, and then centrifuged at 5500 ×g for 10 min. Urine and serum samples were then stored at −80°C for biochemical determinations. The uterus was removed from each rat, cleaned of adhering soft tissues, and immediately weighed. The left femurs were dissected and placed in physiologic saline and stored at −20°C for measurement of bone mineral density (BMD). Right tibias were removed and fixed in 10% neutral-buffered formalin. Mammary glands, vagina, and uteri were removed and each was cut into two parts equally, one part was stored in liquid nitrogen for western blotting analysis, and the other was fixed in 10% neutral-buffered formalin and stored at 4°C.

### 2.4. Assay for Serum and Urine Biochemistry

Serum or urine calcium (Ca), inorganic phosphorus (Pi) concentration, serum alkaline phosphates (ALPs), tartrate resistant acid phosphatase (TRAP), and urine creatinine (Cr) were measured on an automatic analyzer (Ciba-Corning 550, USA) using a diagnostic reagent kit for *in vitro* determination. The levels of estradiol were determined using a specific and sensitive double-antibody RIA kit on a gamma-ray counter. 

### 2.5. Bone Mineral Density Analysis and Bone Histologic Evaluation

The bone mineral density (BMD, g/cm^2^) of the left femur was measured by dual-energy X-ray absorptiometry (LUNAR Co. Ltd., USA) using the small-animal scan mode. Right tibias were then dehydrated in ethanol, defatted in xylene, and embedded and undecalcified in methyl methacrylate. The frontal sections were cut at 4 and 10 *μ*m thicknesses using a microtome (Leica RM 2155, Germany). The 4 *μ*m sections were stained with Goldner's trichrome staining for static histomorphometric measurements; unstained 10 *μ*m sections were used for dynamic histomorphometric analyses by morphometry, which were performed with light and fluorescence microscopes (KSS Computer Engineers, Magna, UT).

### 2.6. Histologic Evaluation of Reproductive Tissues

At the end of treatment, the animals were sacrificed; the uterus, mammary gland, and vagina of each animal were removed. Half of the tissues were collected and immediately fixed in 10% buffered formalin for 48 h. Tissues were then routinely processed in a tissue processor and 5 *μ*m thick sections were prepared and stained with hematoxylin eosin. Histopathologic examination was performed under a light microscope (Leica DMI3000, Germany) and images were analyzed with Image-Pro Plus Software (Media Cybernetics Ltd., USA).

### 2.7. Western Blotting Analysis for ER*α*, ER*β*, and PR Expression

Tissues were homogenized in 50 mL lysis buffer (600 mM Tris-HCl, 1 mM EDTA, pH 7.4, and two protease-inhibitor tablets) with a polytron homogenizer (Kinematics, Luzern, Switzerland). These suspensions were centrifuged for 30 min at 105,00 ×g and the pellet was discarded. The protein concentrations in the supernatants were measured by the method of Bradford. Aliquots containing 50 *μ*g of each sample were precipitated with trichloroacetic acid (TCA, 10% final) and resuspended in 100% methanol. Samples were placed on ice for 20 min and the protein was recovered by centrifugation. Precipitates were dissolved in 1x SDS sample buffer and used for SDS-PAGE. Equal amounts of protein (50 *μ*g) from each sample were loaded per well onto preformed gradient gels, 4–20% acrylamide (NOVEX, San Diego, CA, USA) with a Tris-glycine buffer system. Material was transferred to ProBlot membranes (Applied Biosystems, Foster City, CA, USA), and electroblotting was done in a Tris-glycine buffer. Prestained precision protein standards (Bio-Rad) were used as molecular weight markers. After 60 min of incubation in blocking buffer (10% skim milk in PBS with 0.1% NP-40) at room temperature, membranes were incubated in 1/75 dilution of ER*α*6F11, 1/2500 dilution of ER*β* LBD, or 1/500 dilution of PR*β*2 antibodies in blocking buffer at 4°C, overnight. This was followed by 60 min of washing in blocking buffer and then incubation in 1/7500 dilution of HRP-conjugated secondary antibodies for each species in blocking buffer for 60 min at room temperature. After sequential washing with blocking buffer, PBS with 0.1% NP-40, and PBS, signals were developed using ECL Plus (Amersham Pharmacia Biotech, Uppsala, Sweden). Bands on the exposed film were captured with a Fuji Image Analyzer LAS-1000 and densitometric values were measured with Fuji Image Garge (Fuji Photo Film Co., Japan).

### 2.8. Statistical Analysis

All numerical data were presented as mean ± SD from replicate experiments. One-way analysis of variance followed by Dunnett's *t*-test was used for statistical analysis (PASW 18.0 software; SPSS Inc., Chicago, IL, USA). The significance level was set at *P* < 0.05 for all tests. 

## 3. Results

### 3.1. Body and Uterine Weights

**Figure 1:**
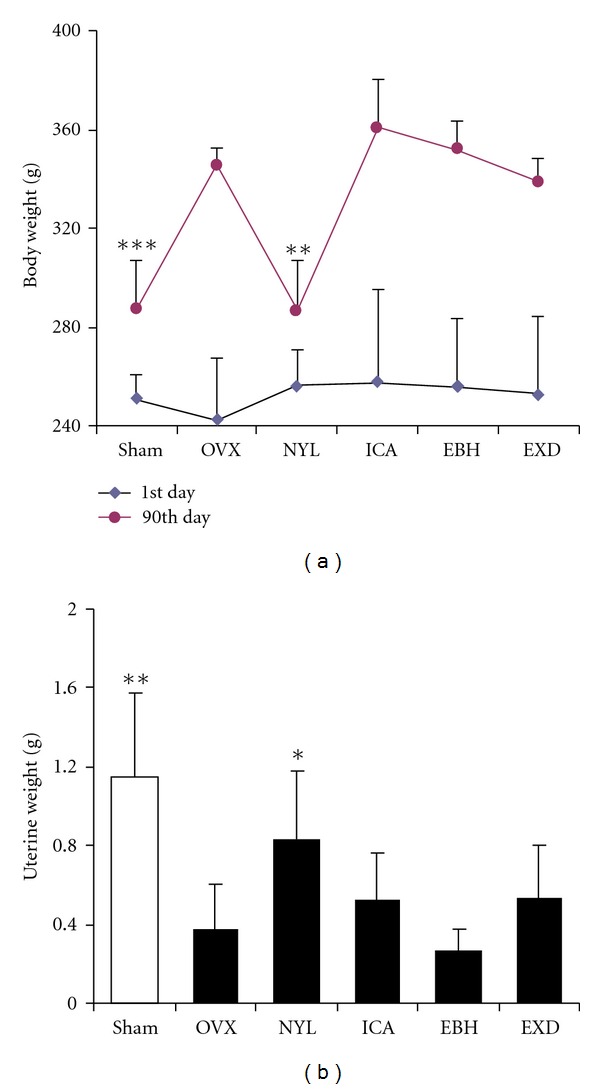
Effects of ICA, EBH, EXD, and NYL on body and uterine weight in ovariectomized osteoporotic rats. Ovariectomized rats were administered NYL (1 mg/kg), ICA (20 mg/kg), EBH (100 mg/kg), or EXD (600 mg/kg) for 12 weeks. The uterine and body weights were weighed. (a): Body weight; (b): uterine weight. Data were presented as mean ± standard deviation, (*n* = 8).  *P < 0.05,  **P < 0.01, and  ***P < 0.001 compared with OVX control.

The rats from all six groups had similar initial mean body weights. At the end of the study, the mean body weight of rats in the OVX group was significantly higher than that of the sham group. NYL treatment completely prevented the increase in body weight associated with E_2_ deficiency, and EXD, EBH, or ICA treatment did not affect the body weight of OVX rats (Figure 1(a)). As expected, the mean uterine weight of OVX animals was significantly lower than that of sham controls, indicating the success of the surgical procedure. Administering NYL significantly increased the uterine weight compared to the OVX group; ICA, EBH, or EXD treatment did not significantly affect the uterine weight in ovariectomized rats (Figure 1(b)). 

### 3.2. Bone Mineral Density and Bone Histological Examination

As shown in Table 1, ovariectomy induced bone loss, as the femoral BMDs of sham and OVX rats were 0.26 ± 0.01 and 0.25 ± 0.01, respectively, indicating that ovariectomy significantly decreased BMD by 5.7% in 12 weeks. The administration of NYL (1 mg/kg), ICA (20 mg/kg), EBH (100 mg/kg), or EXD (600 mg/kg) to the OVX rats significantly increased femoral BMD. The morphologic observations were quantified by histomorphometric analysis of longitudinal cross-sections obtained from the proximal tibiae. Compared with the sham group, there were significant decreases in the percent of trabecular area, trabecular thickness, and trabecular numbers; a significant increase in trabecular separation. The dynamic parameters of mineral rate (MAR), bone formation rate (BFR), and osteoclast number were also significantly increased in OVX rats. The administration of EXD, EBH, ICA, or NYL increased the trabecular area, trabecular thickness, and trabecular numbers in the tibia of OVX rats. However, there were differences in the effects of EXD, EBH, ICA, or NYL on dynamic parameters. NYL and EXD decreased MAR, bone formation rate, and osteoclast number, but ICA and EBH only reduced the osteoclast number and had no effects on bone formation parameters in OVX rats.

### 3.3. Serum and Urine Biochemical Parameters

As shown in Table 2, the measured values for serum calcium (S-Ca) and serum phosphorus (S-P) in rats did not show significant differences among groups, while the ratio of urinary calcium to creatinine (U-Ca/Cr) and the ratio of urinary phosphorus to creatinine (U-P/Cr) levels in the OVX group were significantly higher compared with the sham group, and NYL, EXD, EBH, and ICA significantly prevented the increase in urinary-Ca levels and urinary-P levels in OVX rats. NYL, EXD, EBH, and ICA increased serum estradiol levels in OVX rats; NYL and EXD decreased both ALP and TRAP activities in the serum of OVX rats; only ICA and EBH reduced the TRAP activity, but did not affect the ALP activity in OVX rats.

### 3.4. Histologic Parameters of Uterus, Vagina, and Mammary Gland

**Figure 2:**
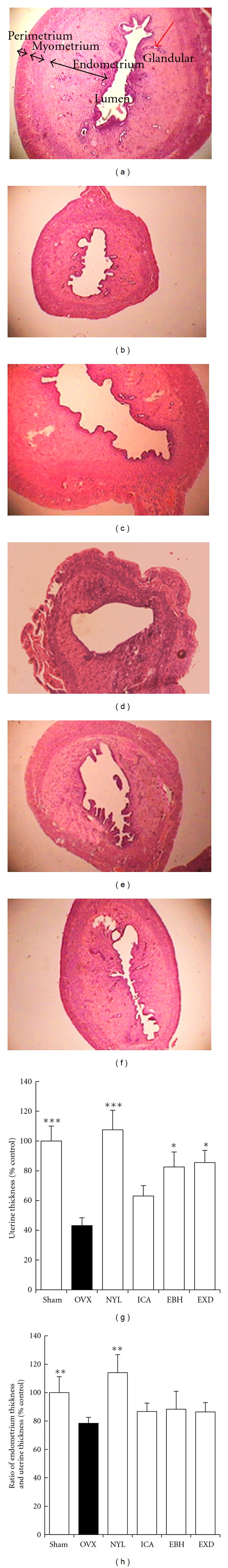
Effects of NYL, ICA, EBH, or EXD on uterine histology in the OVX rats (hematoxylin-eosin staining, magnification ×40). (a): Sham control; (b): OVX control; (c): OVX + NYL treatment; (d): OVX + ICA treatment; (e): OVX + EBH treatment; (f): OVX + EXD treatment; (g): uterine thickness; (h): ratio of endometrial to uterine thickness; the data represent the mean ± standard deviation (*n* = 8);  *P < 0.05;  **P < 0.01 compared with the OVX group.

As shown in Figure 2 and Table 3, uterine lumen in sham-operated rats showed normal endometrial proliferation. In OVX rats, there was a marked decrease in uterine diameter and luminal epithelial cell height. Endometrial stroma was atrophied with a minimal number of glands, the thickness of endometrium and the ratio in uterine thickness were dramatically decreased, and the columnar epithelium of endometrium was fragmented, loose, and thinner. NYL treatment to OVX rats increased markedly uterus, endometrial, luminal epithelium thickness, and endometrial glands numbers; ICA, EBH, or EXD treatment to OVX rats slightly increased the above indices of uterus.

**Figure 3:**
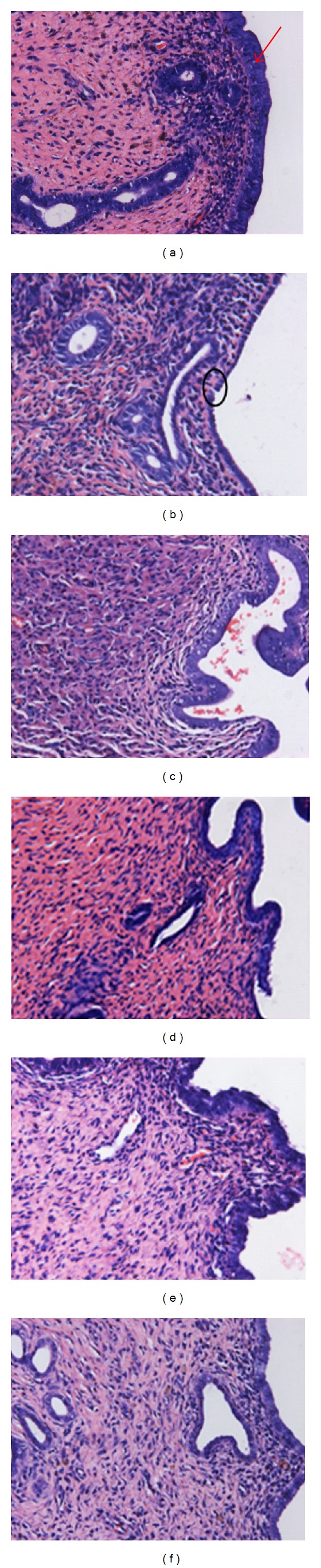
Histologic analysis of uterus (hematoxylin-eosin staining, magnification ×200). (a): Sham group; (b): OVX group; (c): OVX + NYL treatment group; (d): OVX + ICA treatment group; (e): OVX + EBH treatment group; (f): OVX + EXD treatment group.

Figure 3 shows photomicrographs of rat uterine epithelium after treatment. The uteri of sham controls rats were characterized by large cells that formed a tall cuboidal-to-columnar epithelium. That of the OVX control was lined by a low cuboidal epithelium. The uteri of NYL-treated rats demonstrated increased thickness in the endometrial and marked degeneration in the connective tissues, a dramatic increase in myometrial thickness, and a substantial change in the area of the lumen with respect to the entire histologic section. Treatment of OVX rats with ICA, EBH, or EXD slightly increased the uterine epithelial heights.

**Figure 4:**
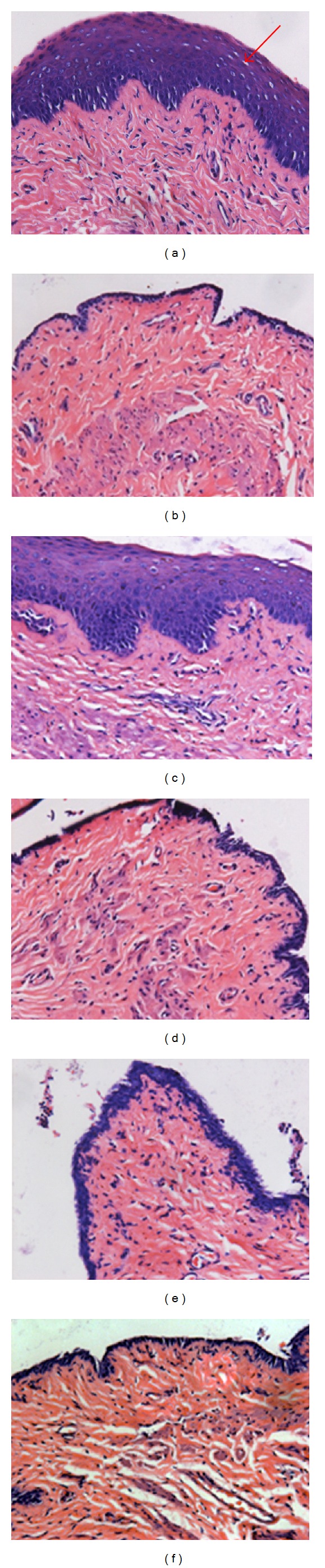
Histologic analysis of vagina (hematoxylin-eosin staining, magnification ×200). (a): Sham group; (b): OVX group; (c): OVX + NYL treatment group; (d): OVX + ICA treatment group; (e): OVX + EBH treatment group; (f): OVX + EXD treatment group.

Figure 4 shows the photomicrographs of the vaginal epithelium of experimental animals after treatment. Compared with sham rats, the vaginal epithelium of OVX rats was atrophic: only two-to-three cell layers were seen, and these were composed of flattened cells with no cornification. Following NYL administration, the diminished vaginal epithelia were reversed, and three layers were observed: the basal stratum germinativum, the intermediate stratum granulosum, and the superficial shedding stratum corneum. Treatment with EXD, EBH, or ICA did not significantly alter vaginal epithelia in OVX rats. NYL treatment increased vaginal epithelial height and cell layers; and treatment with either EXD, EBH, or ICA did not affect the vaginal epithelial heights and cell layers in OVX rats (Table 3).

**Figure 5:**
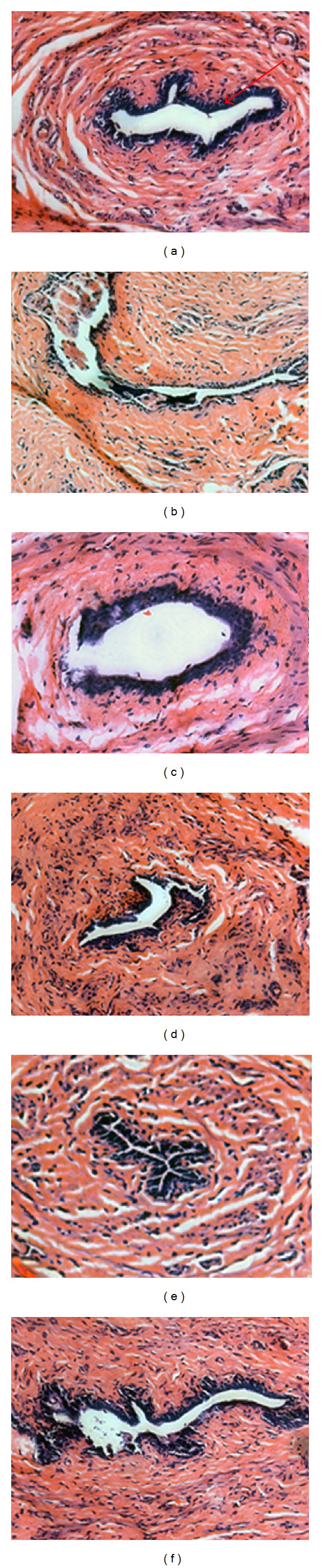
Histologic analysis of mammary gland (hematoxylin-eosin staining, magnification ×200). (a): Sham group; (b): OVX group; (c): OVX + NYL treatment group; (d): OVX + ICA treatment group; (e): OVX + EBH treatment group; (f): OVX + EXD treatment group.

As shown in Figure 5, the mammary glands of rats are composed of connective tissue, acini, and ducts, with the epithelial cells of the acini and ducts manifesting cubic or low columns. The epithelial structures of mammary glands in OVX rats were atrophic, deep in the fat pad, with scarce clusters of densely packed terminal structures, many of which did not show clear luminal formation. Treatment with NYL resulted in abundant terminal epithelial structures. Mammary glands from animals treated with EXD, EBH, or ICA did not differ from those of the OVX controls with respect to the above-mentioned structures. NYL treatment significantly increased mammary gland epithelium thickness; and treatment with either EXD, EBH, or ICA did not affect the mammary gland epithelium thickness in OVX rats (Table 3).

### 3.5. Expression of ER*α*, ER*β*, and PR in Uterus, Vagina, and Mammary Gland

**Figure 6:**
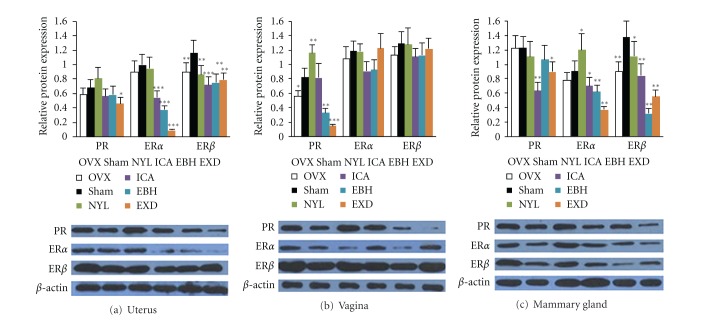
Effects of 12-week ovariectomy treatment and coadministration of NYL, ICA, EBH, and EXD on expression of ER*α*, ER*β*, and PR at protein levels in uterus (a), vagina (b), and mammary gland of rats (c). The protein levels were normalized by *β*-actin. Values are mean ± standard deviation (*n* = 8).  *P < 0.05,  **P < 0.01, and  **P < 0.001 compared with the OVX group.

As shown in Figure 6(a), compared with the sham group, the expression of uterine PR was not significantly different from OVX rats. NYL slightly increased the expression of PR; ICA and EBH slightly decreased the expression of PR; EXD significantly decreased the expression of PR in uteri of OVX rats. Compared with the sham group, the expression of ER*α* was not significantly changed in the uteri of OVX rats; NYL did not change the expression of ER*α*; ICA, EBH, and EXD significantly decreased the expression of ER*α* in the uteri of OVX rats. Compared with the sham group, the expression of ER*β* was significantly increased in the uteri of OVX rats; NYL, ICA, EBH, and EXD significantly decreased the expression of ER*β* in the uteri of OVX rats.

As shown in Figure 6(b), compared with the sham group, the expression of PR in vagina was significantly increased in OVX rats. NYL significantly increased the expression of PR; ICA had no significant effects on the expression of PR; EXD and EBH significantly decreased the expression of PR in the vagina of OVX rats. Compared with the sham group, the expression of ER*α* and ER*β* was not significantly changed; neither NYL, ICA, EBH, nor EXD had any effects on the expression of ER*α* and ER*β* in the vagina of OVX rats.

As shown in Figure 6(c), compared with the sham group, the expression of PR was not significantly changed in the mammary gland of OVX rats. NYL and EBH had no effects on the expression of PR; ICA and EXD significantly decreased the expression of PR in the mammary gland of OVX rats. Compared with the sham group, the expression of ER*α* was slightly increased; NYL significantly increased the expression of ER*α*; ICA, EBH, and EXD significantly decreased the expression of ER*α* in the mammary gland of OVX rats. Compared with the sham group, the expression of ER*β* was significantly increased; NYL slightly decreased the expression of ER*β*; ICA, EBH, and EXD significantly decreased the expression of ER*β* in the mammary gland of OVX rats.

## 4. Discussions

A key goal in therapeutics is to develop clinically effective medicines with superior safety profiles. This study was designed to investigate the potentials of EXD and its major ingredients EBH and ICA on osteoporotic bone and reproductive tissues by comparison with estrogen. The evaluation of the treatments was done with histologic, biochemical, and analyses of ER*α*, ER*β*, and PR expression of uterus, vagina, and mammary gland, in addition to bone mineral density and weight measurements.

Rapid postmenopausal bone loss, which occurs in female rats following ovariectomy, is characterized by a decrease in trabecular bone density and a deterioration of the bone architecture, with a particular diminution in the total number of trabecular and an increase in the number of their perforations. Based on the values of histomorphometric parameters, we found that bone turnover in these OVX rats was increased; EXD, EBH, ICA, or NYL treatment attenuated the deterioration of trabecular bone in the OVX rats. The percentages of trabecular area, thickness, and number were increased, and trabecular separation was decreased in the EXD-, EBH-, ICA-, and NYL-treated OVX rats. The greater bone mineral density and percentage of trabecular area might be due to the increase in both trabecular number and trabecular thickness. EXD or NYL treatment in OVX rats resulted in a decrease in the dynamic parameters for bone resorption and bone formation; EBH or ICA treatment in the OVX rats only led to a significant decrease in the dynamic histomorphometric indices for bone resorption, but not other bone formation parameters. This result suggests that EXD is similar to NYL and exerts a concomitant effect on bone formation and bone resorption at the tissue level, while EBH and ICA produced bone-protective effects mainly by inhibiting bone resorption.

Ovariectomy induces bone loss in rats, leading to an increase in loss of calcium, phosphorous, and creatinine through excretion in urine. Similarly, the increase in levels of ALP and TRAP was observed in the control OVX rats. A reduction in the calcium absorption observed with OVX rats is similar to that for postmenopausal women, and the serum ovarian hormone deficiency can further decrease intestinal calcium absorption [18]. In the present study, EBH and ICA prevented the OVX-induced increase in TRAP activity and urinary calcium and did not affect ALP activity, indicating that EBH and ICA decreased bone loss by inhibiting bone resorption. The characteristics of EXD on bone metabolism are similar to that of Nylestriol. Nylestriol and EXD both significantly decreased serum ALP, TRAP, and urine Ca/Cr levels of OVX rats, indicating that NYL and EXD prevent osteoporosis by inhibiting the high bone turnover through modulating bone formation and bone resorption. The results of our biochemical analyses were found to corroborate the bone histomorphometric results in assessing bone turnover.

The OVX-induced estrogen deprivation was characterized by a significantly increased body weight and decreased uterine wet weight compared to the intact animals. NYL inhibited body weight gain and increased the weight of uteri in OVX rats. EXD, EBH, and ICA did not produce any effects on the treated animals with respect to body and uterine weight, even though EXD, EBH, and ICA were shown to possess estrogenic activity. This lack of uterotrophic activity of EXD, EBH, and ICA could be beneficial in reducing the risk of endometrial, breast, or ovarian cancer associated with estrogen treatment.

The uterus, vagina, and mammary glands are very sensitive to estrogen and represent key organs in clinical safety testing of estrogenic agents. Qualitative analysis of uteri demonstrated increased thickness in the endometrial and marked degeneration in the connective tissues from the NYL-treated rats. Moreover, we observed in this group a dramatic increase in myometrial thickness and a substantial change in the area of the lumen with respect to the entire histologic section, and these uterine phenomena have been reported as typical adverse effects of hormone replacement therapies in women [19]. Thus, the results of the present study substantiate estrogen's stimulatory role in the uterus, vagina, and mammary gland, since there was a significant atrophy in the OVX group. The rats in EXD-, EBH-, and ICA-treated groups maintained a healthy epithelial layer, unlike that of the NYL group.

The endometrial is an exquisitely hormone-sensitive tissue and a principal target of estrogen and progesterone. The coordinated actions of estrogen and progesterone are essential so as to maintain cellular replicative homeostasis. In particular, uterine cell proliferation is augmented by estrogen, which, in turn, is antagonized by progesterone [20, 21]. The effects of estrogen and progesterone on epithelial and endometrial proliferation and differentiation are mediated directly or indirectly through their cognate receptors, namely, estrogen receptor alpha (ER*α*), estrogen receptor beta (ER*β*), and the progesterone receptor (PR) [22]. ER*α* and ER*β* have distinct or even opposing biologic effects in certain cells, where the action of estrogen ligands depends on a balance between ER*α* and ER*β*. In contrast to ER*α*-promoted cancer cell growth, ER*β* inhibits cancer cell proliferation [23, 24]. In endometrial tissue, the expression of PR is known to be ER dependent. In proliferative endometrial and endometrioid carcinoma, PR expression was correlated with ER*α*, but not with ER*β* [25]. ER*α* promotes proliferation, while ER*β* has proapoptotic and prodifferentiating functions. When ER*α* and ER*β* are coexpressed in cells, ER*β* can inhibit the proliferative response of ER*α* on the cells, acting as a dominant inhibitor of ER*α* [24]. In comparison to normal rats producing endogenous estrogen, the expression levels of both estrogen receptor subtypes ER*α* and ER*β* were significantly higher in the OVX rats. This effect described in the literature is a consequence of a negative feedback mechanism [26]. Phytoestrogens interfere with endogenous estrogen action either by acting as agonists when endogenous estrogen levels are low or by acting as antagonists when endogenous estrogen levels are high [27]. The *Epimedium* flavonoids have the peculiar characteristic of a stronger binding action to ER*α*, but with a higher affinity for ER*β* [28]. In our study, there were decreased serum estradiol levels and slightly increased ER*α* and ER*β* expression in uterus, vagina, and mammary gland of OVX rats. NYL treatment induced higher ER*α* and PR expression in uterus, vagina and mammary gland, which caused significant epithelial hyperplasia. The ICA, EBH, and EXD treatments had more potentially inhibitory effects on the expression of ER*α* than on PR and ER*β* expression in uterus and mammary gland of OVX rats, leading to an increase in the ratio of ER*β* to ER*α*. The ratio of ER*β* to ER*α* is associated with decreased stimulatory effects on the endometrium [24]. Therefore, EXD, EBH, and ICA, which have estrogen-like activity, could reduce the bone loss induced by estrogen deficiency and maintain a healthy histomorphology in uterus, vagina, and mammary gland.

EXD is composed of six herbal medicines, with *Epimedium* herbs being its major ingredient. *Epimedium* herbs have been reported to possess abundant flavonoids, including icariin (ICA), epimedin A, B, and C, and hyperin. In animal studies, administering ICA and *Epimedium* flavonoids inhibit bone resorption, prevent osteoporosis in OVX rats [13, 29], and improve erectile function in aged male rats [30]. Icaritin, the aglycon of ICA, has estrogenic properties and stimulates estrogen-driven cells [31]. *Curculigo *rhizomes have been reported to have phenolic glycosides, including curculigoside, curculigoside B and curculigine, and have estrogen-like, antiosteoporotic and antioxidant activity [32]. *Anemarrhena *rhizomes contain steroidal saponin, which are shown to possess estrogenic activity and increase bone formation [33, 34]. *Phellodendron *barks have been shown to have alkaloids, such as berberine and palmatine, which attenuate osteoclast differentiation and function through inhibition of receptor activator nuclear factor-*κ*B ligand in osteoblast cells [35]. *Morinda* root has been reported to have anthraquinone compounds that decrease bone resorption by inhibiting formation and differentiation of osteoclasts induced from bone-marrow cells [36]. The extracts of *Angelica* roots exhibited a stimulation of the uterine histoarchitecture, a significant cornification in the vaginal epithelium, and a reduction in serum luteinizing hormone concentration, thereby exhibiting estrogenic activity [37]. In general, the multiple ingredients of EXD exerted comprehensive effects on bone and estrogen-related organs compared to those of estrogen, but with a fewer adverse effects on the reproductive tissues.

## 5. Conclusions

EXD, EBH, or ICA was as effective as estrogen in restoring the bone density and architecture of ovariectomized rats but caused fewer adverse effects on reproductive tissues. Among the EXD, EBH, and ICA, EXD was found to have superior efficacy and safety profile.

## Figures and Tables

**Table 1:** Mineral density and bone histomorphometric parmeters of proximal tibial cancellous bone.

Groups	Sham	OVX	NYL	ICA	EBH	EXD
BMD (g/cm^2^)	0.26 ± 0.01*	0.25 ± 0.01	0.26 ± 0.01*	0.26 ± 0.01*	0.26 ± 0.01*	0.26 ± 0.01*
Trabecular area ratio (%)	21.37 ± 3.42**	5.32 ± 1.09	19.04 ± 3.28**	10.69 ± 2.22**	13.82 ± 3.71**	18.33 ± 2.47**
Trabecular thickness (*μ*m )	62.42 ± 6.06*	43.57 ± 3.40	63.75 ± 6.39*	56.54 ± 4.72*	54.71 ± 5.16*	60.78 ± 7.02*
Trabecular number (#/mm)	3.17 ± 0.47**	1.31 ± 0.22	2.79 ± 0.31**	1.84 ± 0.24	2.34 ± 0.41**	2.67 ± 0.28**
Trabecular separation (*μ*m )	209.73 ± 40.40**	1007.36 ± 127.63	342.57 ± 37.33**	631.77 ± 58.61*	573.75 ± 62.31*	441.81 ± 51.26*
MAR (*μ*m/d)	0.59 ± 0.19**	1.18 ± 0.24	0.77 ± 0.11*	1.26 ± 0.19	1.08 ± 0.22	0.81 ± 0.19*
BFR/BS (mcm/d*100)	14.62 ± 1.85**	36.88 ± 4.71	17.62 ± 2.02**	31.65 ± 8.76	33.82 ± 4.71	18.23 ± 2.57*
BFR/BV (%/year)	139.62 ± 24.86**	302.55 ± 46.37	163.18 ± 25.72**	314.82 ± 28.66	317.65 ± 47.51	208.39 ± 26.12*
Osteoclast number (#/mm)	7.47 ± 1.25**	18.84 ± 3.67	8.84 ± 2.24**	9.96 ± 2.42**	9.21 ± 1.68**	7.96 ± 2.19**

The data represent the mean ± standard deviation (*n* = 8), *P < 0.05, **P < 0.01 compared with OVX group.

**Table 2:** Osteoporosis-related biochemical indices in different treatment groups.

Groups	Sham	OVX	NYL	ICA	EBH	EXD
S-Ca (mmol/L)	2.80 ± 0.25	2.68 ± 0.21	2.70 ± 0.07	2.56 ± 0.22	2.59 ± 0.18	2.54 ± 0.12
S-P (mmol/L)	2.88 ± 0.55	2.99 ± 0.64	2.61 ± 0.43	3.12 ± 0.74	2.85 ± 0.78	2.61 ± 0.63
U-Ca/Cr	0.40 ± 0.06**	1.29 ± 0.21	0.65 ± 0.11**	0.43 ± 0.05**	0.39 ± 0.06**	0.66 ± 0.08**
U-P /Cr	4.19 ± 0.92*	5.65 ± 1.02	6.03 ± 1.08	3.73 ± 0.90*	3.09 ± 0.87*	2.81 ± 0.90**
Estradiol (pg/mL)	35.71 ± 2.64**	16.73 ± 1.78	28.38 ± 2.23**	20.24 ± 2.12	21.73 ± 2.82*	23.37 ± 1.92*
TRAP (IU/L)	7.22 ± 0.91**	15.01 ± 2.62	3.33 ± 0.54**	4.11 ± 0.63**	3.43 ± 0.84**	3.08 ± 0.73**
ALP (IU/L)	80.59 ± 11.12*	109.02 ± 17.24	85.33 ± 14.61*	104.04 ± 18.71	112.32 ± 19.42	91.82 ± 12.24*

The data represent the mean ± standard deviation (*n* = 8), *P < 0.05, **P < 0.01 compared with OVX group.

**Table 3:** Quantitative data of histological feature in uterus, mammary gland and vagina.

Groups	Sham	OVX	NYL	ICA	EBH	EXD
Uterus thickness (*μ*M)	361.27 ± 38.78***	202.16 ± 16.04	377.41 ± 47.17***	220.31 ± 27.22	248.41 ± 37.35*	253.19 ± 32.23*
Uterus endometrial thickness (*μ*M)	259.15 ± 43.41***	111.42 ± 19.26	290.83 ± 47.11***	128.82 ± 28.17	143.35 ± 41.27	148.45 ± 37.30
Uterus endometrial glands numbers	25.73 ± 2.62***	10.39 ± 1.62	32.14 ± 5.41***	15.83 ± 2.42*	13.14 ± 2.32	16.38 ± 2.71*
Uterus luminal epithelium thickness (*μ*M)	10.62 ± 1.73**	3.94 ± 0.73	9.37 ± 1.92**	3.44 ± 0.61	4.94 ± 0.63	5.25 ± 0.74
Vaginal epithelium cell layers	7.11 ± 2.64***	1.52 ± 0.63	10.28 ± 3.12***	2.09 ± 0.91	2.64 ± 0.72	1.67 ± 0.65
Vaginal epithelium thickness (*μ*M)	23.63 ± 0.81***	4.24 ± 0.41	26.17 ± 1.14***	5.31 ± 0.30	6.12 ± 0.64	4.54 ± 0.83
Mammary gland epithelium thickness (*μ*M)	3.63 ± 0.34**	1.25 ± 0.15	4.64 ± 0.44**	1.14 ± 0.12	1.52 ± 0.21	1.51 ± 0.20

The data represent the mean ± standard deviation (*n* = 8), *P< 0.05, **P < 0.01 compared with OVX group.
